# DFU_DIALNet: Towards reliable and trustworthy diabetic foot ulcer detection with synergistic confluence of Grad-CAM and LIME

**DOI:** 10.1371/journal.pone.0330669

**Published:** 2025-09-02

**Authors:** Monirul Islam Mahmud, Md Shihab Reza, Mohammad Olid Ali Akash, Farhana Elias, Nova Ahmed

**Affiliations:** 1 Department of Electrical and Computer Engineering, North South University, Dhaka, Bangladesh; 2 Design Inclusion and Access Lab, North South University, Dhaka, Bangladesh; Universitas Muhammadiyah Aceh, INDONESIA

## Abstract

Diabetic Foot Ulcer (DFU) is a major complication of diabetes which needs early detection to help in timely treatment for preventing future serious consequences. Due to peripheral neuropathy, high blood glucose levels, and untreated wounds, DFUs can cause the disintegration of the skin and exposing the tissue below it, if not adequately treated. Recently deep learning (DL) has advanced and has shown its ability to automate DFU detection and classification by analysing medical images. The use of DL has been proven to be very useful for healthcare professionals, enabling earlier diagnosis and effective treatment of DFU. However, most of the studies predominantly rely on a single dataset (e.g., DFUC2021 or DFUC2020) without external validation or cross-dataset testing, raising concerns about generalizability and trustworthiness. The aim of this study is to develop a robust, reliable, and transparent DFU detection framework which is not only good performing but also can effectively give attention to the proper region of the images which are crucial for DFU detection. So, to make DFU detection robust, reliable in a single study, we proposed a custom approach, DFU_DIALNet and to enhance transparency and interpret the model decisions in this study, we integrated Grad-CAM and LIME heatmaps to precisely localize ulcer regions. This allows visual verification of the model’s focus and clarifies the decision-making process, thereby increasing the model’s reliability. DFU_DIALNet outperforms all other traditional models with 99.33% accuracy, 99% F1 score, and 100% AUC score, and compared it to other DL models—DenseNet121, MobileNetV2, InceptionV3, EfficientNetB0, ResNet50V2 and VGG16—in the merged dataset of DFUC2021 with our collected 500 images. We have checked our model’s reliability with 2 other popular datasets—-the KDFU and DFUC2020 datasets, where our proposed approach gives the highest accuracy of 95.61% and 99.54%, respectively, compared to other deep learning approaches. Lastly, we have developed a web app using Streamlit to detect DFU efficiently. This study fills the gap between reliable and interpretable systems with a proposed approach to the efficient detection of DFU.

## 1 Introduction

Diabetes is a condition that has an impact on individuals, families, and communities globally. Diabetic foot ulcers (DFU), a common and severe complication of diabetes often leading to limb amputation, are associated with diabetic peripheral neuropathy, caused by microvascular damage, metabolic changes, and a persistent inflammatory state in the axon [53]. According to the International Diabetes Federation, around 463 million people worldwide had diabetes in 2019, with projections indicating this number could reach 700 million by 2045 [1]. Each year more than one million individuals with diabetes undergo leg amputation due to inadequate treatment and identification of diabetic foot ulcers (DFUs) [2,54]. DFUs often occur due to factors like nerve damage, impaired blood flow (vascular issues), and weakened immune system response that are common in people with diabetes. The number of foot ulcers (DFUs) is increasing rapidly; more than a million diabetic patients are at risk of losing part of their foot each year due to a shortage of specialists and inadequate treatment resources [3]. Timely identification and proper classification of foot-related problems enable intervention and effective treatment to heal ulcers. Typically, diagnosis and detection rely on a professional assessment, leaving patients uncertain about whether they have a foot ulcer. Conventional diagnostic approaches require a lot of effort. There is an enhancement in effectiveness and productivity with diagnostic methods compared to the traditional ones. There is always a need for artificial intelligence to detect foot ulcers so that doctors, as well as patients, can be assured about foot ulcers. Traditional methods for analyzing DFU rely on manually crafted techniques [34]. Most of the techniques used for the DFU classification task are conventional machine learning techniques that are sensitive to various sizes, colors, and complicated shapes. In order to effectively detect DFU, numerous studies have been carried out. Various pre-trained models, such as EfficientNet, AlexNet, VGGNet, GoogleNet, MobileNetV2, EfficientNetB0, Inception, ResNet, and NasNet Mobile, have been utilized in research [6,8,24]. Most of the studies relied on common preprocessing techniques, such as data augmentation and segmentation, which were implemented in various studies [5,19]. Different approaches, such as HCNNet and DFU SPNet, have been proposed in studies [20,31]. Additionally, XAI techniques like SHAP and LIME were implemented by researchers [29].

So far, most studies have used classical DL models and proposed different methods that have performed well. Nonetheless, due to the absence of explainability, it is merely infeasible to cross-verify whether their approach correctly identifies the relevant regions of an image for detecting DFU cases. Additionally, they frequently lack generalizability and trustworthiness to ensure consistent and transparent predictions. To address these concerns to mitigate the existing model’s limitations, this study created a merged dataset with DFUC2021 [13] and collected 250 normal and 250 DFU images and proposed a custom approach named DFU_DIALNet that integrates EfficientNetB0 as a feature extractor with a Support Vector Machine (SVM) as the classifier, aiming to enhance the accuracy and efficiency of DFU classification. Accordingly, combining these two offers a better feature representation, with EfficientNetB0 providing a rich feature set capturing essential characteristics necessary for classification, while the SVM constructs decision boundaries capable of handling complex class distributions, leading to improved classification performance. The proposed method was also tested on two other popular datasets, KDFU [18] and DFUC2020 [32], to check its effectiveness and robustness for DFU detection. The datasets were also applied to traditional deep learning (DL) models like DenseNet121, ResNet50, VGG16, InceptionV3, EffecientNetB0, and ResNet50V2 and compared with our proposed approach to evaluate its effectiveness in real life. Then, as an explainable AI (XAI) technique, we have applied Local Interpretable Model-agnostic Explanations (LIME) and Gradient-weighted Class Activation Mapping (Grad-CAM) on the proposed approach to reveal the important mappings to detect DFU images and also visualize the regions that DFU_DIALNet is focusing on, while making predictions, which makes it both more trustworthy and explainable. Lastly, we developed a user-friendly web app with our proposed approach to detect DFU quickly and accurately.

The rest of the paper is organized as follows. Sect 2 provides the overview of the background study, and Sect 3 offers the materials and methods used. Then, Sect 4 gives the result analysis which summarises the findings, and discusses the impact on the field, and Sect 5 shows the conclusion and offers insight for future research directions.

## 2 Related works

A literature review is done to find out what works are currently out there. Several relevant studies have been reviewed, with key contributions from various researchers discussed below.

In the research [5], Moi Hoon Yap et al. used Data augmentation techniques, including HSV and RGB shift, blurring, affine transformation, and brightness adjustment for better detection. In study [27], Ensemble techniques such as NMS, Soft-NMS, and WBF to combine YOLOv8m and FRCNN-ResNet101 are implemented on the DFUC2020 dataset. Mehnoor et al. [9] found that ResNet50 achieves the highest accuracy of 99.49% and 84.76% for Ischaemia and infection, respectively, for the DFU2020 Dataset. However, these studies often overlook systematic comparisons of model complexity, training efficiency, and robustness to diverse clinical conditions, highlighting the need for evaluations of generalizability and real-world deployment constraints. Daniel O. Dantas et al. [35] introduces enhancements to Faster R-CNN by utilizing data augmentation techniques and adjusting parameter settings, and during training, the validation is done by using the Monte Carlo cross-validation technique. In study [14], the proposed method involves extracting texture information from RGB images using mapped binary patterns and feeding both RGB and mapped images to the CNN for DFU recognition.

The DFU_SPNet proposed by Puneeth N. Thotad et al. [6] applied AlexNet, VGGNet, EffecientNet, and GoogleNet where EffecientNet achieved the highest accuracy of 98.97% among all. Ziyang Liu et al. [8] used ResNet, Inception, Ensemble CNN, and EfficientNet to enhance efficiency in wound care, aiding treatment decisions and reducing costs for chronic wounds like DFU. The study of Shiva Shankar Reddy et al. [10] shows that VGG16 is more effective for DFU. In the study [15], the EfficientNet model is converted to a lightweight version with TF Lite for mobile use of DFU detection. CNN model achieves 95% accuracy after training with an 11-layer architecture for at least 70 epochs on a dataset of 1500 DFU images, enabling live predictions in a web application in the study [16]. Aditya Kumar et al. [17] tested a modified ResNet-50 transfer learning model that achieves decent DFU detection. An ensemble model combining ResNet50 and MobileNet with Vision Transformers (ViT), which was introduced in a study conducted by A. A. Pagadala et al. [22]. Laith Alzubaid et al. [33] compared four different hybrid deep convolutional neural network models for DFU classification. In study [26], authors applied Convolutional Neural Networks (CNNs), Feed-Forward Neural Networks (FFNNs), Support Vector Classifiers (SVCs), and Logistic Regression (LR) models to classify DFU images. In the study [7] comparison of ResNet50 and a hybrid model combining ResNet50 with Generative Adversarial Networks (GANs) is shown where The hybrid model outperformed with higher accuracy (0.84). Precision (0.85), and F1-Score (0.84). A proposed DL model, PFUTnet, outperformed Inception V3 and AlexNet, achieving an AUC score of 0.98 and 95% classification accuracy in a study [21]. C. Xie et al. [36] introduced FCFNet, which is a DL framework that involves colour feature extraction using K-Means segmentation and focal loss-based training to improve DFU classification. Shuvo Biswas et al. [37] proposed DFU_MultiNet, which builds on earlier CAD-based DFU detection studies that individually leveraged single pretrained models by utilising transfer learning to fuse multi-scale features from VGG19, DenseNet201 and NasNetMobile via a summing layer. This hybrid approach captures richer representations without manual segmentation, streamlines the workflow by eliminating the need for separate ulcer-region segmentation and achieves superior performance. In Study [38], the impact of visually similar but not identical images in the DFUC2021 dataset on DFU classification performance is studied, and by applying fuzzy similarity algorithms, they find that reducing images with 80% similarity improves classification accuracy with the InceptionResNetV2 model. V. Sendilraj et al. [39] proposed DFUCare as a fully accessible end to end framework for non-invasive DFUs assessment which integrates CIELAB and YCbCr based colour segmentation with YOLOv5s detector and custom DL classifiers. The system achieved a mean average precision (mAP) of 0.861 and an ${\text{F}}_{1}$-score of 0.80 for wound localisation, and demonstrated 79.8 % accuracy in infection prediction and 94.8 % accuracy in ischaemia detection using only standard smartphone images. M. S. A. Toofanee et al. [40] proposed DFU-Helper, which utilises a Siamese Neural Network (SNN) to monitor the progression of DFU over time by computing similarity distances between disease states.This DFU-Helper enables clinicians to evaluate treatment effectiveness and modify care approaches through its combination of pseudo-labeling for anchor threshold refinement and its user-friendly tables and radar charts that display objective progression metrics. R. Karthik et al. [41] proposed an attention-driven DL model named Dense-ShuffleGCANet for DFU classification that consists of DenseNet-169, a Channel-Centric Depth-wise Group Shuffle (CCDGS) block, and triplet attention; the model enhances spatial and cross-channel feature extraction. In study [42], SSODL-DFUDC was introduced by integrating Sparrow Search Optimisation (SSO) with Inception-ResNet-v2 for optimal hyperparameter tuning for DFU detection and classification. In study [43], a DL approach was presented that is based on the pre-trained EfficientNet-B0 architecture for DFU classification using thermographic images. J. Reyes-Luévano et al. [44] introduced a novel visible-infrared deep CNN named DFU_VIRNet for automatic DFU classification and early detection of ulcer risk zones. Bansal, N. et al. [28] introduced DFootNet, which uses multi-sized dense blocks with hierarchical skip connections to extract more complex features and integrated attention layers to achieve both interpretability and high accuracy in DFU classification. Study [45] presents a neural network-based system using an MLP architecture to classify DFU risk as a clinical decision-support tool. S. Wang, J. et al. [4] developed an ML-based model to predict minor amputation needs in patients with University of Texas grade 3 DFU.

Shuvo Biswas et al. [29] developed the DFU_XAI framework to evaluate model interpretability by applying SHAP, LIME and Grad-CAM to Xception, DenseNet121, ResNet50, InceptionV3 and MobileNetV2 architectures. The study shows that ResNet50 achieved the highest performance (98.75 % accuracy, 99.2 % precision, 97.6 % recall, 98.4 % F1-score, 98.5 % AUC) and generated heat maps that endocrinologists found useful for trusting AI diagnostic results. In a study [30] introduces XAI-FusionNet, a multi-scale feature fusion network that distinguishes DFU skin from healthy skin using multiple pre-trained CNNs, a meta-tuner for predictions, and three XAI algorithms for improved transparency. Xie et al. [11] used LightGBM along with 5-fold cross-validation to develop a multi-class classification model for predicting three outcomes of interest, while the SHAP algorithm was employed to interpret the model’s predictions and perform hyperparameter tuning for DFU patients. In study [12], the proposed model extracts features with convolutional networks from a pre-trained VGG16 network and LIME used as an XAI technique for Chronic Wound Classification.

After thoroughly reviewing previous works, it is clear that existing research has yet to fully address the generalizability and interpretability of the models with the end-to-end deployment in a single study. Most methods are trained and evaluated on only one or at best two datasets, leaving real-world robustness untested. The integration of explainability techniques for transparent and clinically trustworthy predictions remains rare among existing approaches, and user-friendly deployment for point of care or remote monitoring is even relatively underexplored. Our proposed DFU_DIALNet framework addresses these gaps by using a merged dataset with collected images for training and also evaluating on two public datasets to ensure reliability while providing Grad-CAM and LIME based visualizations for interpretable heatmaps and local explanations. Lastly, DFU_DIALNet framework is applied on interactive Streamlit based Web application which enables real-time DFU detection.

## 3 Materials and methods

**Fig 1:**
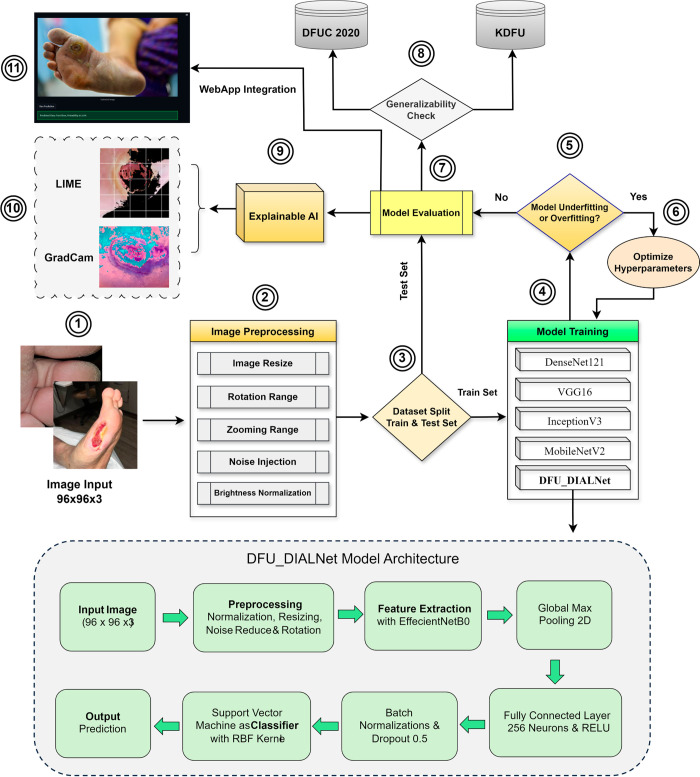
Block diagram of proposed method.

The proposed methods used to detect DFU include six stages: dataset details. Data preprocessing, models & evaluation, Proposed DFU DIALNet Architecture, explainability of models, and experimental setup. Fig 1 illustrates overall work overflows.

### 3.1 Dataset details

**Fig 2:**
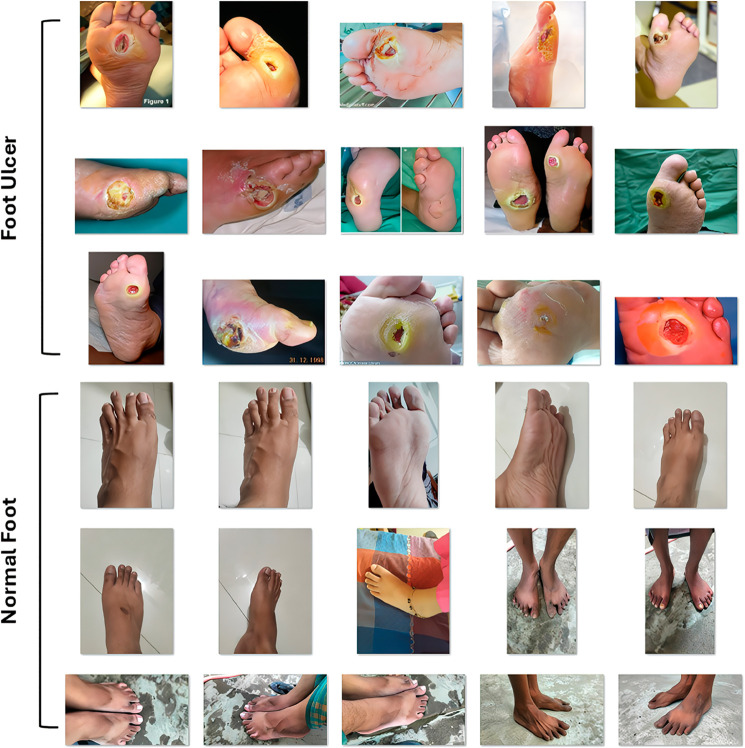
Normal and Ulcer foot images in merged DFUC 2021.

**Fig 3:**
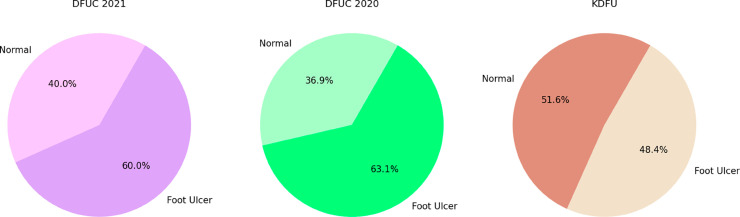
Data Distribution of DFUC2021, DFUC2020 and Kaggle DFU Datasets.

This study combines 500 images, 250 diabetic foot ulcer images collected from private hospitals in Dhaka and Chittagong, Bangladesh, and 250 healthy foot images obtained from individual volunteers in the same region, added with the Foot Ulcer Challenge “DFUC2021” [13] dataset. So, the total number of images in our merged dataset is 3000 now. Although this approach does not completely balance the dataset, it reduces the imbalance. During data collection the acquisition of all 500 DFU images occurred through a manual smartphone under consistent indoor lighting conditions against a non-reflective neutral background. The system disabled auto-exposure and auto-white-balance functions while applying indoor white balance and mid-level exposure settings to all images to reduce variability. The images received immediate anonymisation following their capture. The data collection process was conducted from $5^{\text{th}}$ December 2024 to $8^{\text{th}}$ January 2025, approved by the Institutional Review Board (IRB)/Ethics Review Committee (ERC) of North South University, Bangladesh (Application No. 2024/OR-NSU/IRB/1110). During data collection, an informed written consent form was obtained where the data provider was explicitly informed that the data would be used solely for this research. The data will remain confidential and will not be publicly available, and all necessary security measures will be maintained. Further for the generalisability check, we utilized the KDFU [18] and DFUC 2020 [32] datasets. Fig 2 shows the sample picture of the hybrid dataset and Fig 3 shows the data distribution visualization of DFUC 2021, DFUC 2020, and the KDFU Datasets. Table 1 shows the dataset distribution of all datasets.

**Table 1:** Dataset distribution.

Dataset	Train Set		Test Set	
	Ulcer	Normal	Ulcer	Normal
DFUC 2021 [13]	1350	800	450	400
Kaggle DFU [18]	512	543	153	167
DFUC 2020 [32]	2000	1500	2000	843

### 3.2 Data preprocessing

Data preprocessing plays a critical role in enhancing input image data quality, mitigating overfitting, and improving model generalization. This is particularly vital when integrating collected data with external datasets to construct hybrid datasets, as well as when ensuring robust model performance across diverse datasets during evaluation. To harmonize the characteristics of images from our collected dataset (500 images) and the DFUC2021 dataset Our study employed an extensive array of preprocessing techniques to achieve the inherent variability of clinical imaging summarized in Table 2. All images were first resized to 96×96×3, in line with the DFUC2021 dataset, where input size standardization helps decrease computational complexity while maintaining important pathological details. Rotations up to 15^°^ were used in the preprocess to consider differences in patient positioning and the angular differences that are part of the image acquisition. Moreover, a zoom factor of 1.15 was used to simulate scale and distance differences and expand the model’s capability of detecting DFUs irrespective of their size and proximity. To simulate sensor noise and other possible imaging artifacts Gaussian noise (with mean 0 and variance 0.01) was added to the input, forcing the network to learn that features are invariant to these artifacts. In addition, brightness standardization with factor 1.1 was applied to correct the lighting fluctuations. To maintain clinical realism and avoid the annotation burden and potential pre-processing errors introduced by multi-stage detection–segmentation pipelines, lesion segmentation was not performed [55,56]. The same preprocessing pipeline, including resizing (96x96x3 pixels), rotations, zoom, noise injection, and brightness adjustment, was applied to the DFUC2020 and KDFU datasets. These static augmentation parameters were selected to reflect clinically observed variability while avoiding unrealistic distortions and applying consistent steps across all sources to ensure reproducibility and minimize source-specific bias. By eliminating differences in resolution and acquisition protocols, this approach guarantees that the model learns features truly invariant to imaging conditions. As a result, domain shifts between datasets are minimized, enabling the model to generalize effectively to unseen data while maintaining diagnostic fidelity.

**Table 2:** Preprocessing and data augmentation techniques with parameters.

Number	Augmentation Technique	Parameter Values	Purpose
1	Image Resizing	(96×96×3)	Standardizes input size for compatibility with DL models.
2	Rotations	$15^{\circ }$	Enhances robustness to positional variations.
3	Zooming Factor	1.15	Aids generalization by introducing slight variations.
4	Gaussian noise	Mean = 0, Variance = 0.01	Improves model robustness against adversarial perturbations.
5	Brightness Standardization	Factor: 1.1	Standardizes illumination variations to improve model robustness and ensure consistent feature extraction.

### 3.3 Models and evaluation

We have split the dataset into 80% (Train Set) and 20% (Test Set), which ensures that most of the available data are utilized for training while leaving enough data to reliably evaluate the model, which also ensures the robustness with fewer overfitting issues in the comparatively small dataset. For datasets that are not extremely large, this ratio provides a good trade-off between training and testing. After dividing the dataset correctly, we analyzed some Deep Learning models which previously demonstrated superior performance in DFU detection such as DenseNet121, MobileNetV2, VGG16, InceptionV3, EffecientNetB2, ResNet50V2 [46–49] and our proposed hybrid model, named ‘DFU_DIALNet’. The training process included class-weighted loss functions to handle class imbalance without modifying data, following established practice for imbalanced medical image classification tasks [57] The training process assigned higher penalties to minority class misclassifications which reduced the bias toward the majority class. A model checkpoint is used in the training process to resume where it left off rather than beginning anew in the case of training being interrupted for any cause, including hardware failure. After implementing all the models, we evaluated these models using Accuracy, Precision, Recall, F1 Score, Specificity and AUC curve. We also plotted the Confusion Matrix for all the models which assesses how well the categorization models perform in terms of making predictions on test data and provides an overall rating of our model’s performance.

The DenseNet121 [46] model comes pre-loaded, with weights trained on the ImageNet dataset forming the foundation for feature extraction. To tailor it for a classification task, custom layers are integrated into the existing DenseNet121 model in this study, such as a Global Average Pooling layer that condenses each feature map into a value while preserving information, a Dense layer with 1024 units and ReLU activation and an Output Dense layer with units corresponding to the number of classes. The model is compiled using the Adam optimizer, a sparse categorical cross-entropy loss function. The MobileNetV2 [47] model comes equipped with trained weights sourced from the ImageNet dataset. We also added the Global Average Pooling Layer, Dense Layer, and Output Dense Layer as custom layers in MobileNetV2. The InceptionV3 [49] model comes equipped with existing weights derived from the ImageNet dataset. The model is put together using the Adam optimizer and sparse categorical cross-entropy loss function. The VGG16 model [48] comes equipped with existing weights sourced from the ImageNet dataset. In this research, the VGG16 model incorporates a global average pooling layer, a dense layer with ReLU activation and 1024 units, and an output dense layer with SoftMax activation. The EfficientNetB0 [50] model uses compound scaling to uniformly scale depth, width, and resolution with a set of fixed scaling coefficients. Pre-trained on ImageNet, we integrate a global average pooling layer to reduce spatial dimensions, followed by a dense layer of 1024 units with ReLU activation and a final output dense layer matching the number of DFU classes with SoftMax activation. The ResNet50V2 [55] model uses pre-activation residual units by placing batch normalization and ReLU activation before each convolutional layer. The model begins with ImageNet-trained weights, followed by a Global Average Pooling layer, then Dense layers with 1,024 units and ReLU activation, and finally an Output Dense layer with SoftMax activation. All the model’s parameter are shown in Table 3. The training and evaluation pipeline for all models follows the same protocol, ensuring a fair comparison of their performance in this study.

**Table 3:** Model parameters.

Model	Parameters
DenseNet121	Optimizer: Adam; Loss: sparse_categorical_crossentropy; Dense units: 1024; Activation: ReLU, Softmax; Weight init: ImageNet
InceptionV3	Optimizer: Adam; Loss: sparse_categorical_crossentropy; Dense units: 1024; Activation: ReLU, Softmax; Weight init: ImageNet
MobileNetV2	Optimizer: Adam; Loss: sparse_categorical_crossentropy; Dense units: 1024; Activation: ReLU, Softmax; Weight init: ImageNet
VGG16	Optimizer: Adam; Loss: sparse_categorical_crossentropy; Dense units: 1024; Activation: ReLU, Softmax; Weight init: ImageNet
EffecientNetB0	Optimizer: Adam; Loss: binary_crossentropy; Batch size: 32; Activation: Swish, Sigmoid; Weight init: GlorotUniform
ResNet50V2	Optimizer: Adam; Loss: binary_crossentropy; Activation: ReLU, Sigmoid; Weight init: HeNormal (Conv)
DFU_DIALNet	Learning rate: 5e-4; Optimizer: Adam; Batch size: 64; Loss: sparse_categorical_crossentropy; Dropout: 0.5; Dense units: 256; Activation: ReLU, Softmax; Regularization: BatchNormalization; Weight init: ImageNet

### 3.4 Proposed DFU_DIALNet architecture

In this study, we have proposed a custom approach, “DFU_DIALNet” which consists of EfficientNetB0 and SVM models. EfficientNetB0 is used for the feature extractor, and the SVM model is used as a classifier. Although we leverage EfficientNetB0’s deep-CNN architecture to extract hierarchical features, classification is performed by SVM; this hybrid is often termed deep feature-based machine learning. The EfficientNetB0 model comes with trained weights sourced from the ImageNet dataset. To maintain these trained weights and prevent them from being updated during training, the layers of the EfficientNetB0 model are kept frozen. Its convolutional layers study spatial hierarchies of dataset features by slowly converting input images into more complex ones. It is the output of the convolutional layer and the weights and biases, where X represents the input, and then the ensuing convolutional process of EfficientNetB0, executed levels of a layer, can be presented as in [23]:

$$y_{conv}=f(W_{conv}*X+b_{conv})$$ (1)

Custom layers are then added on top of this model including a Global Max Pooling Layer, a Dense Layer with 256 units, and ReLU activation to capture patterns, the outputs of the layer. Global Max Pooling [23] is an operation performed on every feature map to shrink the spatial dimensions and generate a fixed-length vector that has the only most informative features:

$$Y_{pool}=\max_{i,j}\;Y_{conv}\;(i,j)$$ (2)

$$Y_{dense}=\text{ReLU}\left(W_{dense}\cdot Y_{pool}+b_{dense}\right)$$ (3)

The model is optimized using the Adam optimizer with a learning rate set at 0.0005 and a dropout layer with a rate of 0.5 while employing the cross-entropy loss function. Training spans 200 epochs on the training data which is validated using test data in batches of size 64. This Dense layer [23] refines the feature representation using the ReLU activation function. An SVC is trained using a basis function (RBF) kernel based on features extracted from the training data with the EffecientNetB0 model. The processed feature vectors are then directed toward the Support Vector Classifier (SVC) using a Radial Basis Function (RBF) kernel, which is a powerful tool for handling nonlinearly separable data. As per [24], the RBF kernel function is an integral factor of the SVC and depicts the similarity between feature vectors x and x’ in this way:

$$K(x,x^{\prime })=\exp(-\gamma {\left\|x-x^{\prime }\right\|}^{2})$$ (4)

where *γ* refers to the flexibility of the kernel and at the same time, the locality of the feature contribution depends on this parameter. The Kubernetes domain now becomes the feature space, and in this way, the SVC achieves a successful partitioning of the intricate class boundaries through the kernel. The SVC decision function [25] that is described as follows:

$$f(x)=\sum_{i=1}^{n}\alpha_{i}y_{i}\;K(x,x^{\prime })+b$$ (5)

**Fig 4:**
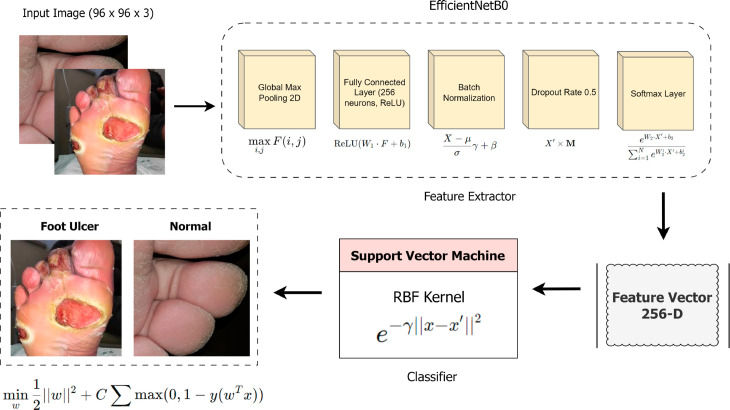
Architecture of proposed DFU_DIALNet approach.

The model’s performance is evaluated through accuracy and loss metrics offering insights into its efficacy across categories. Lastly, the DFU_DIALNet model is based on the EfficientNetB0 of the deep and layered feature extraction method together with SVM. EfficientNetB0 is selected as a lightweight backbone due to its strong accuracy to complexity trade-off, achieving high performance with significantly fewer parameters and reduced computational overhead to detect DFU more accurately [50–52] We paired it with SVM because SVM is known for its robust decision boundaries in high-dimensional spaces, particularly effective when dealing with relatively small and imbalanced datasets like ours. Additionally, SVMs are less prone to overfitting and offer better generalization with limited data. The proposed DFU_DIALNet algorithm based on this work has been summarized in Algorithm 1 and Fig 4 shows the architecture of DFU_DIALNet.

**Algorithm 1. Pseudocode of proposed DFU_DIALNet approach.**

**Require:** $X_{\text{train}}\in {\mathbb{R}}^{n\times d}$: training features, $Y_{\text{train}}\in \{0,1{\}}^{n}$: training labels,

$X_{\text{test}}\in {\mathbb{R}}^{m\times d}$: test features, $Y_{\text{test}}\in \{0,1{\}}^{m}$: test labels,

*θ*: termination threshold for training error.

**Ensure:** Pred: Predicted labels, Acc: Classification accuracy.

1: Initialize EfficientNet with weights 𝒲: ℱ←EfficientNet(X;𝒲)

2: Initialize 𝒲 using He normal or Xavier initialization

3: **repeat**

4:   Forward pass: $\hat{Y}=\sigma (ℱ(X_{\text{train}};𝒲))$

5:   Compute loss: $ℒ=-\frac{1}{n}\sum_{i=1}^{n}\left[y_{i}\log({\hat{y}}_{i})+(1-y_{i})\log(1-{\hat{y}}_{i})\right]$

6:   Backpropagation: $𝒲\leftarrow 𝒲-\eta \cdot \nabla_{𝒲}ℒ$, where learning rate *η*

7:   error←ℒ

8: **until** error<θ

9: ${\text{Features}}_{\text{opt}}\leftarrow \text{Select}(X_{\text{train}},Y_{\text{train}})$

10: Extract top hidden layer features: $H_{\text{train}}\leftarrow {ℱ}_{\text{top}}(X_{\text{train}})$

11: Concatenate deep and selected features: $Z_{\text{train}}=[H_{\text{train}}\|{\text{Features}}_{\text{opt}}]$

12: Train SVM: Solve the following optimization:


$$\min_{𝐰,b,\xi }\frac{1}{2}\|𝐰{\|}^{2}+C\sum_{i=1}^{n}\xi_{i}\;\text{s.t.}\;y_{i}({𝐰}^{⊤}\phi (z_{i})+b)\geq 1-\xi_{i},\;\xi_{i}\geq 0$$

where $z_{i}\in Z_{\text{train}}$, ϕ(·) is the kernel mapping, and *C* is the penalty parameter.

13: $H_{\text{test}}\leftarrow {ℱ}_{\text{top}}(X_{\text{test}})$

14: $Z_{\text{test}}=[H_{\text{test}}\|{\text{Features}}_{\text{opt}}]$

15: $\text{Pred}\leftarrow {\text{SVM}}_{\text{predict}}(Z_{\text{test}})$

16: $\text{Acc}\leftarrow \frac{1}{m}\sum_{i=1}^{m}𝕀(y_{i}={\hat{y}}_{i})$

17: **Grad-CAM:** For class *c*, compute importance weights for feature map ${\text{A}}^{\text{k}}$:


$$\alpha_{k}^{c}=\frac{1}{Z}\sum_{i}\sum_{j}\frac{\partial y^{c}}{\partial A_{ij}^{k}}$$

$${\text{GradCAM}}^{c}=\text{ReLU}\left(\sum_{k}\alpha_{k}^{c}A^{k}\right)$$

18: **LIME:** For an input *x*, generate perturbed samples $x^{\prime }\in 𝒵$ and predict $f(x^{\prime })$:


$$\text{LIME}(x)=\arg\min_{g\in G}ℒ(f,g,\pi_{x})+\Omega (g)$$

19: **Return** (Pred,Acc)

### 3.5 Explainability of models

Explainable artificial intelligence (XAI) enables humans to understand and trust the results and output produced by machine learning algorithms. Gradients of a specific target that flow through the convolutional network are used by Grad-CAM to locate and highlight target regions in the image. Guided Grad-CAM produces high-resolution detail of the target class in an image by fusing Grad-CAM with pre-existing pixel-space gradient visualizations. Grad-CAM cannot emphasize fine-grained details, despite being class discriminative and able to identify significant image regions. To get around this, high-resolution visualizations are created by fusing the Grad-CAM and Guided Backpropagation images using element-wise multiplication. By negating the gradient of yc with respect to feature maps A of a convolutional layer, one can force the network to alter its predictions. As a result, the important weights are now:

$$\alpha_{k}^{c}=\frac{1}{z}\sum_{i}\sum_{j}\left(-\frac{\partial y^{c}}{\partial A_{i,j}^{k}}\right)$$ (6)

By learning an interpretable model locally around the prediction, LIME provides an accurate and interpretable explanation for any classifier’s prediction. LIME focuses on explaining the model’s prediction for specific instances rather than offering a comprehensive knowledge of the model across the entire dataset. The result consists of three primary pieces of information: (1) the model’s predictions; (2) the contributions of the features; and (3) the actual value for each feature. The process of finding a simple, interpretable surrogate model *g* that approximates the complex model *f* locally around a point of interest, weighted by πκ, which emphasizes locality, then a formal representation of the optimization objective used in LIME [26]:

$$\epsilon (x)=\arg\min L(f,g,\pi_{x})+\Omega (g)$$ (7)

### 3.6 Experimental setup

- **Hardware:** For this research work, we used Intel 13th Gen Core i9 CPU, 128 GB of RAM, 16GB NVIDIA RTX A4000, and 4TB NVMe M.2 SSD.
- **Software:** This project uses the Windows 11 OS, Python, TensorFlow, Scikit-learn and Jupyter Notebook (Executed with Local CPU/GPU support).

## 4 Result analysis and discussion

The performance of DFU_DIALNet, the proposed approach, was compared against six baseline DL models: DenseNet121, MobileNetV2, InceptionV3, VGG16, EfficientNetB0, and ResNet50V2, as shown in Tables 4 and 5.

**Table 4:** Result evaluation of all models in DFUC2021 dataset.

Model Evaluation Result			
Model	Train Accuracy	Test Accuracy	Test Loss
DenseNet121	100%	97.67%	0.10
MobileNetV2	99.17%	64.00%	2.45
InceptionV3	98.83%	96.67%	0.06
VGG16	97.37%	93.00%	0.19
EfficientNetB0	98.79%	98.00%	0.08
ResNet50V2	99.37%	96.50%	0.13
DFU_DIALNet	100%	99.33%	0.02

**Table 5:** Classification report of different models in DFUC2021 dataset.

Classification Report								
Model	Precision		Recall		Specificity		F1 Score	
	Ulcer	Normal	Ulcer	Normal	Ulcer	Normal	Ulcer	Normal
DenseNet121	1.00	0.96	0.95	1.00	1.00	0.95	0.98	0.98
MobileNetV2	0.58	1.00	1.00	0.28	0.28	1.00	0.74	0.44
InceptionV3	0.98	0.97	0.97	0.98	0.98	0.97	0.98	0.98
VGG16	1.00	0.88	0.86	1.00	1.00	0.86	0.92	0.93
EfficientNetB0	0.97	0.98	0.98	0.97	0.98	0.97	0.98	0.98
ResNet50V2	0.99	0.94	0.94	0.99	0.93	0.99	0.96	0.97
DFU_DIALNet	1.00	0.99	0.99	1.00	1.00	0.99	0.99	0.99

**Fig 5:**
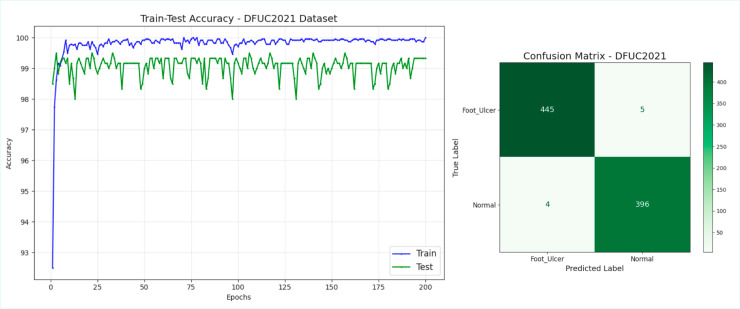
Train vs Test accuracy plot and Confusion Matrix of DFU_DIALNet in DFUC2021.

Moreover, it observed an excellent performance in DFU detection with a test accuracy and test loss of 99.33% and 0.02 on the DFUC2021 dataset, respectively, achieving superior generalization and robustness.While DenseNet121 and InceptionV3 both achieved a 97.67% accuracy (test loss 0.10 and 0.06, respectively), EfficientNet-b0 matched this performance with 97.67% accuracy (test loss 0.08), and ResNet50V2 reached 96.50% accuracy (test loss 0.13); VGG16 and MobileNetV2 performed poorly by only achieving an accuracy of 93% and 64%, respectively. DFU_DIALNet reached almost perfect precision, recall, and F1 scores (1.00, 0.99, and 0.99), while MobileNetV2 performed poorly (F1 score: 0.74) for ulcers, highlighting its failure to classify complex cases reliably. EfficientNetB0 achieved a precision of 0.97 for ulcers and 0.98 for normals, recall of 0.98 and 0.97, specificity of 0.98 and 0.97, and F1 scores of 0.98 for both classes. ResNet50V2 showed strong performance with precision of 0.99 (ulcer) and 0.94 (normal), recall 0.94 and 0.99, specificity 0.93 and 0.99, and F1 scores of 0.96 (ulcer) and 0.97 (normal). In terms of specificity across both classes, DFU DIALNet achieves the highest overall specificity, 1.00 for the ulcer class and 0.99 for the normal class, making it the best performer in terms of specificity among all models. The architecture of DFU_DIALNet effectively extracted both global and local features, addressing challenges like class imbalance in medical datasets. With significantly high recall values for either class of ulcer and normal, it laid out minimum false discoveries and provided reliable predictions. Despite the lower test losses, the recall for ulcers was low for VGG16, while adequate, could not attain the proper balance between recalls and precision and achieve a lower F1 score of 0.92 for ulcers. Fig 5 shows the Train vs. Test accuracy plot and the confusion matrix for the DFU_DIALNet model in the DFUC2021 dataset.

The learning curves also proved the robustness of DFU_DIALNet during training and validation over 200 epochs. This shows that the model is able to generalize very well because the training accuracy stabilized at 100%, however the testing accuracy stabilized around 99.33%. The DFU_DIALNet confusion matrix shows that the model correctly identified 445 foot ulcer samples and 396 normal samples out of a total of 850 samples. This results in just 9 misclassifications, five false negatives, and four false positives. This result indicates that almost all of the images from the testing dataset are classified correctly by DFU_DIALNet, which is crucial in medical diagnostics that require accurate detection of ostensibly small anomalies such as foot ulcers for timely and effective treatment.

### 4.1 Reliability check of DFU_DIALNet approach

**Fig 6:**
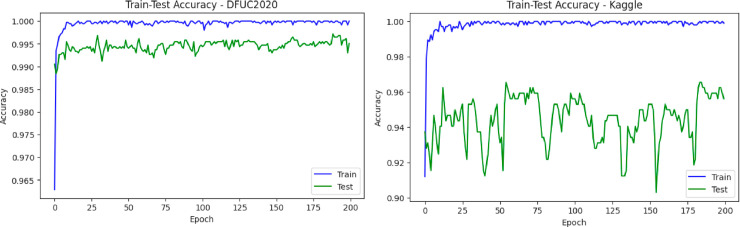
Performance evaluation of DFU_DIALNet: Train vs Test Accuracy plot in DFUC2020 and KDFU Dataset.

**Fig 7:**
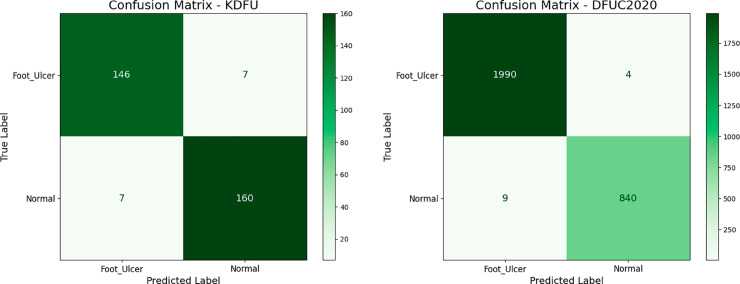
Confusion Matrix of DFU_DIALNet in KDFU & DFUC2020 dataset.

The reliability of the DFU_DIALNet model was assessed through its performance on two benchmark datasets: the KDFU dataset [18] and the DFUC2020 dataset [32]. Table 6 shows the training and testing accuracy, test loss, precision, recall, specificity, and F1-score as key performance metrics. On the KDFU dataset, DFU_DIALNet achieved 99.91 % training accuracy and 95.61 % testing accuracy, with a test loss of 0.21; precision, recall, F1 score were all 0.96, and specificity was 0.95. On the DFUC2020 dataset, DFU_DIALNet reaches a training accuracy of 100% and a test accuracy of 99.54% with a very low test loss of 0.009. Precision, recall, specificity, and F1 score were all over 0.99, highlighting that the model is also handling more complicated data very well. Figs 6 and 7 shows train vs. test accuracy and confusion matrices for both datasets, thus enabling a holistic perspective of the model for classification accuracy.

**Table 6:** Result Evaluation of DFU_DIALNet model on KDFU and DFUC2020 datasets.

Result Evaluation						
Dataset	Accuracy (%)	Test Loss	Precision	Recall	specificity	F1 Score
KDFU [18]	95.61	0.21	0.96	0.96	0.95	0.96
DFUC2020 [32]	99.54	0.009	0.99	0.99	0.99	0.99

**Fig 8:**
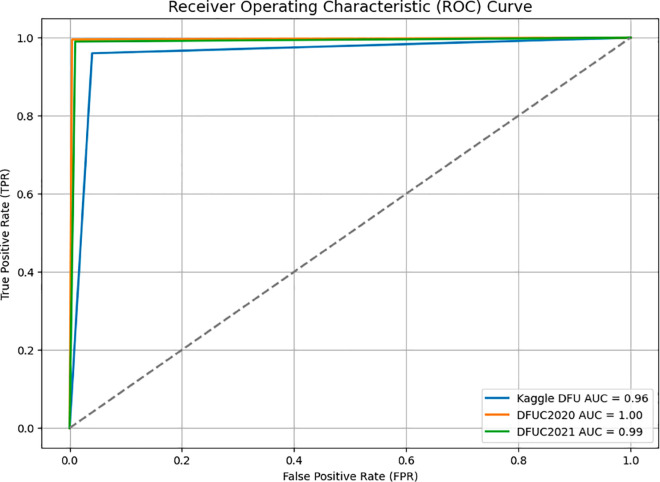
Receiver Operating Characteristic curve of DFU_DIALNet in DFUC2021, KDFU & DFUC2020 datasets.

**Fig 9:**
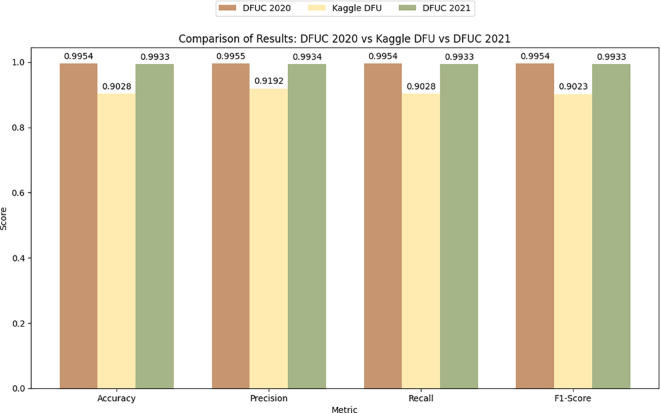
Result Comparison of DFU_DIALNet in DFUC2021, KDFU & DFUC2020 datasets.

Fig 8 shows the Receiver Operating Characteristic (ROC) curve of DFU_DIALNet on the DFUC2021, KDFU, and DFUC2020 datasets, with corresponding Area Under Curve (AUC) values of 0.99, 0.96 and 1.00, respectively. While DFU_DIALNet achieves an AUC of 1.00 on the DFUC2020 dataset, representing perfect classification, also achieves an exceptionally near-perfect AUC score of 0.99 on the DFUC2021 dataset. The AUC of 0.96 on the KDFU dataset was only slightly lower than on the validation datasets, which suggests similarly good model performance in diverse datasets. Fig 9 shows the result comparison of DFU_DIALNet in DFUC2021, KDFU & DFUC2020 dataset. The superior performance of DFU_DIALNet in diabetic foot ulcer (DFU) classification stems from its hybrid architecture, which merges EfficientNetB0’s scalable feature extraction with RBF kernel and SVM’s discriminative power through cross-dataset validation. The compound scaling mechanism of EfficientNetB0 extracts both large-scale foot anatomy details and small-scale ulcer texture information. The ImageNet pretrained weights of EfficientNetB0 remain frozen to maintain robust multi-level filters which prevent overfitting when working with limited DFU images. The Global Max Pooling layer transforms these features into essential descriptors, which the 256-unit ReLU block with dropout and statistical selection refines to generate compact, information-dense embeddings. The RBF SVM head functions as a replacement for softmax establishes maximal margin non-linear boundaries, which provides better performance in handling class imbalance and detecting ulcers. The dual-stream input architecture of EfficientNet embeddings and saliency-selected features allows the model to represent both anatomical and pathological details. Table 7 presents a comparison of several research studies that utilised the DFUC2021, KDFU & DFUC2020 datasets alongside different approaches with trustworthiness and generalisability which further validated DFU_DIALNet across multiple datasets, enhancing its generalisation as a DFU classification solution.

**Table 7:** Comparison of performance across various research studies and proposed DFU_DIALNet approach on the DFUC2021, KDFU & DFUC2020 datasets.

Performance Comparison					
Study	Dataset	Technique	Best Performance	Explainable AI (XAI)	Generalizability Check
Mehnoor et al. [9]	DFUC2020 [32]	ResNet50	Accuracy of 99.49% for Ischaemia 84.76% and infection.	×	×
Bansal, N et al. [28]	KDFU [18]	DFootNet	Accuracy of 98.87%.	×	×
Biswas, S et al. [29]	KDFU [18]	ResNet50	Accuracy of 98.75%.	×	×
C. Xie et al. [36]	DFUC2021 [13]	EfficientNet B3 and ResNeXt50	Accuracy of 69.97% and infection	×	×
Shuvo Biswas et al. [37]	KDFU [18]	VGG19, DenseNet201, and NasNetMobile.	Accuracy of 99.06%	×	×
I. C. Dipto et al. [38]	DFUC 2021 [13]	Inception ResNet V2	Accuracy of 62.1%	×	×
V. Sendilraj et al. [39]	DFUC2020 [32] & DFUC 2021 [13]	DenseNet121	Accuracy of 94.81%	×	✓
M. S. A. Toofanee et al. [40]	DFUC 2021 [13] & KDFU [18]	DFU-Helper with Siamese Neural Network (SNN)	F1 Score of 0.76	×	✓
R. Karthik et al. [41]	DFUC2021 [13]	Dense-ShuffleGCANet	Accuracy of 86.09%	×	×
S. Nagaraju et al. [42]	KDFU [18]	SSODL-DFUDC	Accuracy of 99.29%	×	×
P. Shanmugam et al. [43]	KDFU [18]	EfficientNet-B0 trained on thermographic images for DFU classification.	Accuracy of 98.71%	×	×
J. Reyes-Luévano et al. [44]	KDFU [18] & 4 Collected datasets	DFU_VIRNet with GAP-2D-DLSA-IMG substructure.	0.9121 (AUC) and 0.8363 (F1-score) for Infection classification.	×	✓
**This Study**	**KDFU [18], DFUC 2020 [32], DFUC 2021 [13]**	**Proposed DFU_DIALNet approach, consisting of EfficientNetB0 as Feature Extractor and SVM as Classifier.**	**Accuracy of 99.54% in DFUC2020, 95.61% in KDFU, and 99.33% in DFUC2021 datasets.**	✓	✓

### 4.2 Explainability with Grad-Cam & LIME

**Fig 10:**
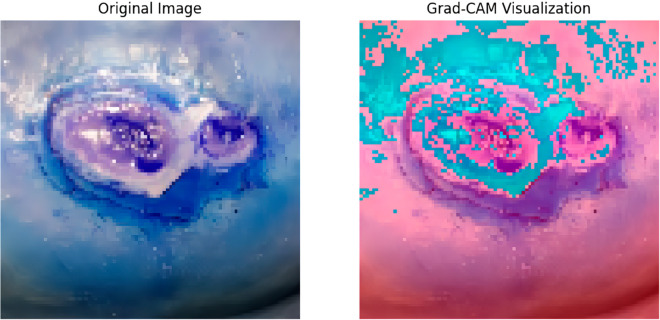
GradCam Explanation with DFU_DIALNet.

**Fig 11:**
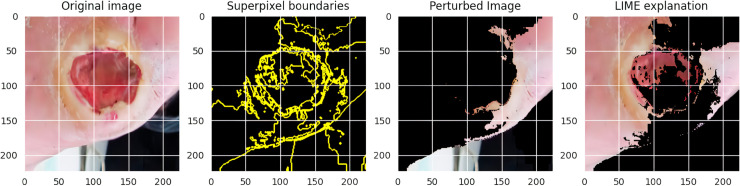
LIME Explanation with DFU_DIALNet.

From our study, we need to ensure that, although our proposed DFU_DIALNet performs well, the model’s decision-making process must be explainable. This is important to verify that our approach is correctly identifying the regions of an image to detect DFU images, which is crucial for the model’s reliability and trustworthiness. We examined a randomly selected image from our dataset to illustrate both XAI techniques, namely GradCam and LIME, which are depicted in Fig 10 and Fig 11, respectively. Grad-CAM visualizations showing image regions that are given importance for classification by DFU_DIALNet. Heatmap overlay on the input image highlighting regions of high (cyan) and low (magenta) contribution. As we can see on the heatmap above, the model clearly focuses on the ulcerated center of the image, as well as clinically relevant areas that are most important when diagnosing DFU. The correlation of these regions with the physical characteristics of the ulcer suggests that the model learns predictive visual patterns, such as irregular edges and discoloration, to make predictions. The LIME visualization also presents a detailed and comprehensible analysis of the DFU_DIALNet model’s decision-making process, revealing the image regions that played a significant role in its classification.

The original image illustrates a diabetic foot ulcer with key clinical features such as tissue discoloration and irregular texture. The Superpixel Boundaries panel divides the image into superpixel regions, which are subsequently used to analyze the local model predictions. Using these superpixels, the perturbed image simulates variations by selectively masking certain regions to assess their influence on the model’s output. The resulting LIME explanation highlights the superpixels most critical to the classification, with the highest-contributing regions overlaid on the original image. In this LIME explanation, we can see that the model mostly relies on the ulcerated part and its surroundings while making decisions, as well as other things such as irregular edges, texture abnormalities, and color contrast. By visualizing the explanations, LIME not only shows the model’s focus on particular critical image areas but also enhances trustworthiness.

**Fig 12:**
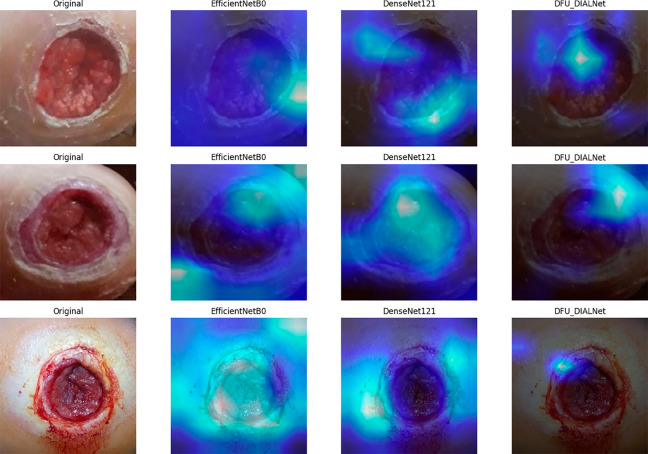
GradCam Visualization of high performing models in Ulcer Foot images.

Moreover, to evaluate the performance of the DFU_DIALNet, we compared the performance of EfficientNet-b0 and DenseNet121 (as these are the second and third highest accuracy holders) by taking three randomly selected DFU images from the testing data as shown in GradCAM visualizations in Fig 12 to see which regions of the image each model focuses on. The heatmaps highlight the regions of focus for each model in making a prediction. The Grad-CAM heatmaps show that DFU_DIALNet’s attention maps are tightly focused on ulcer-centric regions, accurately mirroring clinical ground truth in the original images. In contrast, EfficientNet-B0 and DenseNet121 produce diffuse or misaligned heatmaps that often highlight peripheral textures, healthy skin, or imaging artifacts. For example, DenseNet121 may fixate on non-ulcer edges, while EfficientNet-B0 overemphasizes homogeneous areas, both reflecting poor localization of pathological features. Although the raw accuracy metrics of EfficientNet-B0 and DenseNet121 are comparable to those of DFU_DIALNet, their explanations reveal misaligned attention regions during decision-making. DFU_DIALNet consistently concentrates high-intensity activations around lesion regions, confirming its superior capability for DFU-related feature extraction. By comparison, EfficientNet-B0 and DenseNet121, despite achieving close accuracy, frequently direct attention toward peripheral skin textures, background shading, or irrelevant corners. DFU_DIALNet’s Grad-CAM heatmaps clearly highlight only the ulcer area, focusing on the color, tissue growth, and edges that clinicians use to judge wound severity.

**Fig 13:**
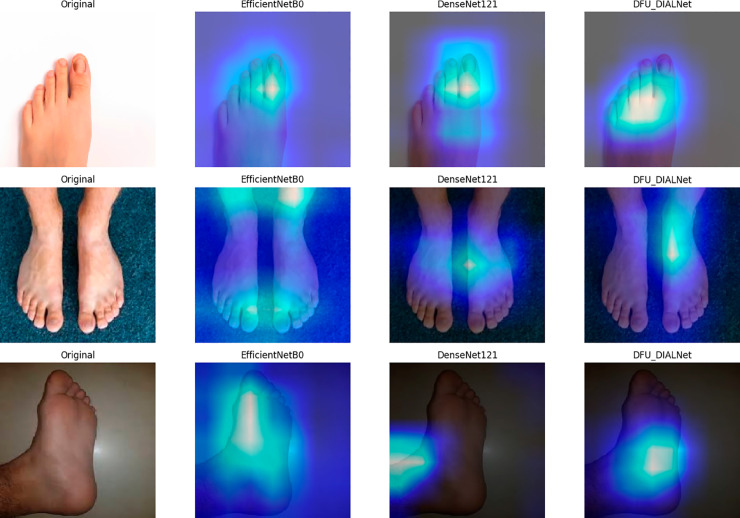
GradCam Visualization of high performing models in Normal Foot images.

To verify that our model’s predictions depend on anatomically relevant features rather than on confounding background elements, we applied Grad-CAM to the “normal foot” class, as shown in Fig 13. The visualizations show that EfficientNetB0 and DenseNet121 focus their attention on non-anatomical parts such as image borders and floor textures, but DFU_DIALNet focuses its heatmaps only on clinically significant areas, including the plantar surface and toe joints and uniform skin regions, while ignoring background artifacts. The Grad-CAM overlays demonstrate that DFU_DIALNet activates its attention specifically on the anatomical parts of the foot (toes, ball, and arch). The background areas received significant attention from EfficientNetB0 and DenseNet121 while they failed to focus on relevant parts. The focused activation pattern demonstrates that DFU_DIALNet uses actual anatomical cues for its “healthy foot" predictions instead of image features that are not relevant.

### 4.3 WebApp integration

**Fig 14:**
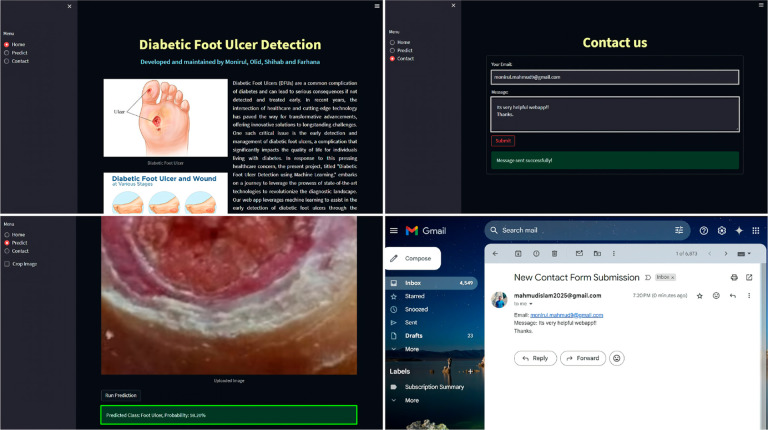
Developed WebApp to detect DFU images.

In this study, we developed a web app with the DFU_DIALNet model using Streamlit, a Python library, which is hosted on Render (https://footulcer-bd.onrender.com). The web app has a prediction page, where users can upload the foot image and contact page to get user feedback. The app predicted the image of an ulcer foot with 98.20% confidence, as represented in Fig 14, suggesting the app’s potential for deployment in healthcare and research activities in the real world. For a low-income country, where medical treatment and detection of foot ulcers are much more costly, this free-of-cost web app can be a blessing and promising technology, reducing the burden on the health sector.

## 5 Conclusion and future work

Computational methods have been pivotal in driving much advancement in both the detection and prevention of DFUs in recent years. In this work, we proposed DFU_DIALNet, a custom approach that consists of EfficientNetB0 as a feature extractor with a Support Vector Machine (SVM) as the classifier for DFU detection and classification, and compared its performance against traditional DL models, including DenseNet121, MobileNetV2, InceptionV3, and VGG16. The DFU_DIALNet was found to produce exceptionally high results with a test accuracy of 99.33%, a low test loss of 0.02, and near-perfect precision, recall, and F1 scores in DFUC2021. DFU_DIALNet not only achieves higher accuracy but also has the ability to fully capture characteristics of DFU, as it focuses on regions that are highly correlated with DFU, where traditional DL models may fall short. The model demonstrated generalisability across diverse popular DFU datasets, including KDFU and DFUC2020, indicating the robustness of the DFU_DIALNet approach to different data distributions. The integration of explainable AI methods like Grad-CAM and LIME improved the interpretability and explainability of the model, aiding trust in the clinical setting. Further, Grad-CAM visualization revealed that compared to other models, our proposed approach, DFU_DIALNet, is correctly identifying the regions of normal or ulcer foot images to correctly detect DFU. Also, a simple web app is created based on this model, which can be used to predict if an image represents a healthy foot or one with DFU. Yet limitations do exist with our research study. At present, it only processes image data from users, ignoring all other relevant clinical information, such as the patient’s medical history, comorbidities, and demographics, that could make this research work even more accurate and relevant in real life. Secondly, DFU_DIALNet demonstrates strong performance, but further evaluation is needed in resource-constrained settings. Future directions, however, should be to enlarge the dataset by adding more normal and foot ulcer images from other populations to help overcome these limitations. Optimizing efficiency and validating performance on edge devices for real-world clinical use will be explored through knowledge distillation to enable efficient edge deployment testing of DFU_DIALNet. Moreover, future studies could optimize the model’s performance by integrating multimodal data, including electronic health records (EHR) data and sensor data, which would improve the model’s prediction and generalisability in diverse healthcare settings. Ultimately, the incorporation of DFU_DIALNet into real-time diagnostic systems may also allow for the deployment of early DFU detection, which can support clinical decision-making, improve patient management, and reduce severe complications and adverse outcomes in diabetic foot patients.

## References

[1] Ogurtsova K, da Rocha Fernandes JD, Huang Y, Linnenkamp U, Guariguata L, Cho NH, et al. IDF Diabetes Atlas: global estimates for the prevalence of diabetes for 2015 and 2040. Diabetes Res Clin Pract. 2017;128:40–50. doi: 10.1016/j.diabres.2017.03.024 28437734

[2] Armstrong DG, Lavery LA, Harkless LB. Validation of a diabetic wound classification system. The contribution of depth, infection, and ischemia to risk of amputation. Diabetes Care. 1998;21(5):855–9. doi: 10.2337/diacare.21.5.855 9589255

[3] Swerdlow M, Shin L, D’Huyvetter K, Mack WJ, Armstrong DG. Initial clinical experience with a simple, home system for early detection and monitoring of diabetic foot ulcers: the foot selfie. J Diabetes Sci Technol. 2023;17(1):79–88. doi: 10.1177/19322968211053348 34719973 PMC9846401

[4] Wang S, Wang J, Zhu MX, Tan Q. Machine learning for the prediction of minor amputation in University of Texas grade 3 diabetic foot ulcers. PLoS One. 2022;17(12):e0278445. doi: 10.1371/journal.pone.0278445 36472981 PMC9725167

[5] Yap MH, Hachiuma R, Alavi A, Brüngel R, Cassidy B, Goyal M, et al. Deep learning in diabetic foot ulcers detection: a comprehensive evaluation. Comput Biol Med. 2021;135:104596. doi: 10.1016/j.compbiomed.2021.104596 34247133

[6] Thotad PN, Bharamagoudar GR, Anami BS. Diabetic foot ulcer detection using deep learning approaches. Sensors International. 2023;4:100210. doi: 10.1016/j.sintl.2022.100210

[7] El-Kady AM, Abbassy MM, Ali HH, Ali MF. Advancing diabetic foot ulcer detection based on ResNet and GAN integration. Journal of Theoretical and Applied Information Technology. 2024;102(6):2258–68.

[8] Liu Z, John J, Agu E. Diabetic foot ulcer ischemia and infection classification using efficientnet deep learning models. IEEE Open J Eng Med Biol. 2022;3:189–201. doi: 10.1109/ojemb.2022.3219725 36660100 PMC9842228

[9] Ahsan M, Naz S, Ahmad R, Ehsan H, Sikandar A. A deep learning approach for diabetic foot ulcer classification and recognition. Information. 2023;14(1):36. doi: 10.3390/info14010036

[10] Reddy SS, Alluri L, Gadiraju M, Devareddi R. Forecasting diabetic foot ulcers using deep learning models. Lecture Notes in Networks and Systems. Singapore: Springer; 2023. p. 211–27. 10.1007/978-981-19-7874-6_16

[11] Xie P, Li Y, Deng B, Du C, Rui S, Deng W, et al. An explainable machine learning model for predicting in-hospital amputation rate of patients with diabetic foot ulcer. Int Wound J. 2022;19(4):910–8. doi: 10.1111/iwj.13691 34520110 PMC9013600

[12] Sarp S, Kuzlu M, Wilson E, Cali U, Guler O. The enlightening role of explainable artificial intelligence in chronic wound classification. Electronics. 2021;10(12):1406.

[13] Yap MH, Cassidy B, Kendrick C. Diabetic foot ulcers grand challenge. Springer; 2022. 10.1007/978-3-030-94907-5

[14] Al-Garaawi N, Ebsim R, Alharan AFH, Yap MH. Diabetic foot ulcer classification using mapped binary patterns and convolutional neural networks. Comput Biol Med. 2022;140:105055. doi: 10.1016/j.compbiomed.2021.105055 34839183

[15] Mulyono S, Chalimah Sadyah NA, Much Ibnu Subroto I, Chaerul Haviana SF, Satrio Waluyo Poetro B, Syaifuddin NM, et al. Classification of ischemic, infectious, and normal diabetic foot ulcers based on the EfficientNet model. In: 2023 10th International Conference on Electrical Engineering, Computer Science and Informatics (EECSI). 2023. p. 7–11. 10.1109/eecsi59885.2023.10295901

[16] Gayatri G, Varun AS, Sai MJ, Chowdari ChP, Jamal K. Diabetic foot ulcer identification using machine learning. In: 2023 International Conference on Sustainable Computing and Data Communication Systems (ICSCDS). 2023. p. 309–15. 10.1109/icscds56580.2023.10104696

[17] Kumar A, Nelson L, Singh S. ResNet-50 transfer learning model for diabetic foot ulcer detection using thermal images. In: 2023 2nd International Conference on Futuristic Technologies (INCOFT). 2023. 10.1109/incoft60753.2023.10425447

[18] Diabetic foot ulcer (DFU). Kaggle. 2021. https://www.kaggle.com/datasets/laithjj/diabetic-foot-ulcer-dfu

[19] Giridhar C, Akhila B, Kumar SP, Sumalata GL. Detection of multi stage diabetes foot ulcer using deep learning techniques. In: 2024 3rd International Conference on Applied Artificial Intelligence and Computing (ICAAIC). 2024. p. 553–60. 10.1109/icaaic60222.2024.10575186

[20] Das SK, Namasudra S, Sangaiah AK. HCNNet: hybrid convolution neural network for automatic identification of ischaemia in diabetic foot ulcer wounds. Multimedia Systems. 2024;30(1). doi: 10.1007/s00530-023-01241-4

[21] Sharma N, Mirza S, Rastogi A, Mahapatra PK, Kumar D. PFUTnet: a novel deep learning architecture for diabetic foot severity mapping and analysis. IEEE Sensors J. 2024;24(9):14770–7. doi: 10.1109/jsen.2024.3377212

[22] Pagadala AA, Silas S, Joy E. Ensemble of vision transformers and CNNs for accurate diabetic foot ulcer classification. In: 2024 International Conference on Cognitive Robotics and Intelligent Systems (ICC - ROBINS). 2024. p. 300–4. 10.1109/icc-robins60238.2024.10533993

[23] Demirkol D, Erol ÇS, Tannier X, Özcan T, Aktaş Ş. Prediction of amputation risk of patients with diabetic foot using classification algorithms: a clinical study from a tertiary center. Int Wound J. 2024;21(1):e14556. doi: 10.1111/iwj.14556 38272802 PMC10789580

[24] Arnia F, Saddami K, Roslidar R, Muharar R, Munadi K. Towards accurate diabetic foot ulcer image classification: leveraging CNN pre-trained features and extreme learning machine. Smart Health. 2024;33:100502. doi: 10.1016/j.smhl.2024.100502

[25] Fadhel MA, Alzubaidi L, Gu Y, Santamaría J, Duan Y. Real-time diabetic foot ulcer classification based on deep learning & parallel hardware computational tools. Multimed Tools Appl. 2024;83(27):70369–94. doi: 10.1007/s11042-024-18304-x

[26] Sweta SR. Advanced machine learning techniques for diabetic foot ulcer detection. Journal of Electrical Systems. 2024;20(10s):63–71.

[27] Sarmun R, Chowdhury MEH, Murugappan M, Aqel A, Ezzuddin M, Rahman SM, et al. Diabetic foot ulcer detection: combining deep learning models for improved localization. Cogn Comput. 2024;16(3):1413–31. doi: 10.1007/s12559-024-10267-3

[28] Bansal N, Vidyarthi A. DFootNet: a domain adaptive classification framework for diabetic foot ulcers using dense neural network architecture. Cogn Comput. 2024;16(5):2511–27. doi: 10.1007/s12559-024-10282-4

[29] Biswas S, Mostafiz R, Paul BK, Uddin KMM, Hadi MdA, Khanom F. DFU_XAI: a deep learning-based approach to diabetic foot ulcer detection using feature explainability. Biomedical Materials & Devices. 2024;2(2):1225–45. doi: 10.1007/s44174-024-00165-5

[30] Biswas S, Mostafiz R, Uddin MS, Paul BK. XAI-FusionNet: diabetic foot ulcer detection based on multi-scale feature fusion with explainable artificial intelligence. Heliyon. 2024;10(10):e31228. doi: 10.1016/j.heliyon.2024.e31228 38803883 PMC11129011

[31] Das SK, Roy P, Mishra AK. DFU_SPNet: a stacked parallel convolution layers based CNN to improve Diabetic Foot Ulcer classification. ICT Express. 2022;8(2):271–5. doi: 10.1016/j.icte.2021.08.022

[32] Cassidy B, Reeves ND, Pappachan JM, Gillespie D, O’Shea C, Rajbhandari S, et al. The DFUC 2020 dataset: analysis towards diabetic foot ulcer detection. touchREV Endocrinol. 2021;17(1):5–11. doi: 10.17925/EE.2021.17.1.5 35118441 PMC8320006

[33] Alzubaidi L, Abbood A, Fadhel M, Al-Shamma O, Zhang J. Comparison of hybrid convolutional neural networks models for diabetic foot ulcer classification. Journal of Engineering Science and Technology. 2021;16:2001–17.

[34] Wang Y, Luo F, Yang X, Wang Q, Sun Y, Tian S, et al. The swin-transformer network based on focal loss is used to identify images of pathological subtypes of lung adenocarcinoma with high similarity and class imbalance. J Cancer Res Clin Oncol. 2023;149(11):8581–92. doi: 10.1007/s00432-023-04795-y 37097394 PMC11796498

[35] Oliveira A, Britto de Carvalho A, Dantas D. Faster R-CNN approach for diabetic foot ulcer detection. In: Proceedings of the 16th International Joint Conference on Computer Vision, Imaging and Computer Graphics Theory and Applications, 2021. p. 677–84. 10.5220/0010255506770684

[36] Xie C. FCFNet: a network fusing color features, focal loss for diabetic foot ulcer image classification. In: Tanveer M, Agarwal S, Ozawa S, Ekbal A, Jatowt A and editors. Neural Information Processing. ICONIP 2022. Communications in Computer and Information Science, vol 1793. Singapore: Springer; 2023. p. 438–49. 10.1007/978-981-99-1645-0_36

[37] Biswas S, Mostafiz R, Paul BK, Mohi Uddin KM, Rahman MM, Shariful FNU. DFU_MultiNet: a deep neural network approach for detecting diabetic foot ulcers through multi-scale feature fusion using the DFU dataset. Intelligence-Based Medicine. 2023;8:100128. doi: 10.1016/j.ibmed.2023.100128

[38] Chen P, Lou G, Wang Y, Chen J, Chen W, Fan Z, et al. The genetic basis of grain protein content in rice by genome-wide association analysis. Mol Breed. 2022;43(1):1. doi: 10.1007/s11032-022-01347-z 37312871 PMC10248653

[39] Sendilraj V, Pilcher W, Choi D, Bhasin A, Bhadada A, Bhadadaa SK, et al. DFUCare: deep learning platform for diabetic foot ulcer detection, analysis, and monitoring. Front Endocrinol (Lausanne). 2024;15:1386613. doi: 10.3389/fendo.2024.1386613 39381435 PMC11460545

[40] Toofanee MSA, Dowlut S, Hamroun M, Tamine K, Duong AK, Petit V, et al. DFU-helper: an innovative framework for longitudinal diabetic foot ulcer diseases evaluation using deep learning. Applied Sciences. 2023;13(18):10310. doi: 10.3390/app131810310

[41] Ajay A, Singh Bisht A, Karthik R. Dense-ShuffleGCANet: an attention-driven deep learning approach for diabetic foot ulcer classification using refined spatio-dimensional features. IEEE Access. 2025;13:5507–21. doi: 10.1109/access.2024.3524549

[42] Nagaraju S, Kumar KV, Rani BP, Lydia EL, Ishak MK, Filali I, et al. Automated diabetic foot ulcer detection and classification using deep learning. IEEE Access. 2023;11:127578–88. doi: 10.1109/access.2023.3332292

[43] Shanmugam P, Manoj Kumar PK, Hussain MM, R Revathi, Deepa P, Sakthivelu U. A deep learning model-based neural network framework for diabetic foot ulcer classification. In: 2024 5th International Conference on Electronics and Sustainable Communication Systems (ICESC). 2024. p. 1305–10. 10.1109/icesc60852.2024.10690106

[44] Reyes-Luévano J, Guerrero-Viramontes JA, Rubén Romo-Andrade J, Funes-Gallanzi M. DFU_VIRNet: a novel visible-InfraRed CNN to improve diabetic foot ulcer classification and early detection of ulcer risk zones. Biomedical Signal Processing and Control. 2023;86:105341. doi: 10.1016/j.bspc.2023.105341

[45] Ferreira ACBH, Ferreira DD, Barbosa BHG, Aline de Oliveira U, Aparecida Padua E, Oliveira Chiarini F, et al. Neural network-based method to stratify people at risk for developing diabetic foot: a support system for health professionals. PLoS One. 2023;18(7):e0288466. doi: 10.1371/journal.pone.0288466 37440514 PMC10343027

[46] G A, R PR, G S, D P. Tailored deep learning approaches for binary classification and evaluation of diabetic foot ulcer images. In: 2024 Third International Conference on Intelligent Techniques in Control, Optimization and Signal Processing (INCOS), 2024. p. 1–7. 10.1109/incos59338.2024.10527575

[47] Arnia F, Saddami K, Muharar R, Dwi Pratiwi DA, Nurdin Y. Diabetic foot ulcer detection on mobile platforms through thermal imaging and deep learning. In: 2023 International Conference on Smart-Green Technology in Electrical and Information Systems (ICSGTEIS). 2023. p. 104–8. 10.1109/icsgteis60500.2023.10424364

[48] Güley O, Pati S, Bakas S. Classification of infection and ischemia in diabetic foot ulcers using VGG architectures. Diabet Foot Ulcers Grand Chall 2021. 2022;13183:76–89. doi: 10.1007/978-3-030-94907-5_6 35465060 PMC9026672

[49] Geeitha S, Aravinth S, Rishikesh K, Nishanth J, Renuka P. Diabetes foot ulcer detection using inception V3 deep learning technique. In: 2024 10th International Conference on Advanced Computing and Communication Systems (ICACCS). 2024. p. 899–904. 10.1109/icaccs60874.2024.10717009

[50] Yap MH, Cassidy B, Pappachan JM, O’Shea C, Gillespie D, Reeves ND. Analysis towards classification of infection and ischaemia of diabetic foot ulcers. In: 2021 IEEE EMBS International Conference on Biomedical and Health Informatics (BHI). 2021. 10.1109/bhi50953.2021.9508563

[51] Verma G. Leveraging smart image processing techniques for early detection of foot ulcers using a deep learning network. Pol J Radiol. 2024;89:e368–77. doi: 10.5114/pjr/189412 39139256 PMC11321030

[52] Almufadi N, Alhasson HF. Classification of diabetic foot ulcers from images using machine learning approach. Diagnostics (Basel). 2024;14(16):1807. doi: 10.3390/diagnostics14161807 39202295 PMC11353632

[53] Putra MIA, Gusti N, Duta TF, Alina M, Qanita I, Naufal MA, et al. Vitamin D supplementation improves foot ulcers among diabetic patients: Pooled analysis of randomized controlled trials. Narra X. 2023;1(3). doi: 10.52225/narrax.v1i3.104

[54] Margalin B, Arfijanto MV, Hadi U. Effector function and neutrophil cell death in the severity of sepsis with diabetes mellitus. Narra J. 2024;4(1):e532. doi: 10.52225/narra.v4i1.532 38798871 PMC11125301

[55] Rahimzadeh M, Attar A. A modified deep convolutional neural network for detecting COVID-19 and pneumonia from chest X-ray images based on the concatenation of Xception and ResNet50V2. Inform Med Unlocked. 2020;19:100360. doi: 10.1016/j.imu.2020.100360 32501424 PMC7255267

[56] Dhar MK, Wang C, Patel Y, Zhang T, Niezgoda J, Gopalakrishnan S, et al. Wound tissue segmentation in diabetic foot ulcer images using deep learning: a pilot study. arXiv preprint 2024. https://arxiv.org/abs/2406.16012

[57] Haibo He, Garcia EA. Learning from imbalanced data. IEEE Trans Knowl Data Eng. 2009;21(9):1263–84. doi: 10.1109/tkde.2008.239

